# 

**Published:** 1906-11-15

**Authors:** 


### CONTENTS.

Event and Comment - - - - - - - - - - 541

The Necessity for Reform in Dental Education,
By D. D. Smith, D.D.S., M.D. 542

Indents - - - - - - - - - - - 583

Announcements - - - - - - - - - - 588

